# Functional outcomes following nerve transfers for shoulder and elbow reanimation in brachial plexus injuries: a 10-year retrospective study

**DOI:** 10.25122/jml-2025-0079

**Published:** 2025-04

**Authors:** Cristian-Vladimir Vancea, Florin-Vlad Hodea, Eliza-Maria Bordeanu-Diaconescu, Stefan Cacior, Catalina-Stefania Dumitru, Vladut-Alin Ratoiu, Alexandru Stoian, Ioan Lascar, Dragos Zamfirescu

**Affiliations:** 1 Department 11, Discipline of Plastic and Reconstructive Surgery, Carol Davila University of Medicine and Pharmacy, Bucharest, Romania; 2 Clinic of Plastic Surgery and Reconstructive Microsurgery, Clinical Emergency Hospital of Bucharest, Bucharest, Romania; 3 Clinic of Plastic Surgery and Reconstructive Microsurgery, Zetta Hospital, Bucharest, Romania; 4 Clinic of Plastic Surgery and Reconstructive Microsurgery, Sf. Ioan Emergency Clinical Hospital, Bucharest, Romania

**Keywords:** brachial plexus palsy, nerve transfers, nerve reconstruction, shoulder/elbow function restoration

## Abstract

Brachial plexus injuries are rare but highly disabling, with major implications for upper limb function and quality of life. Nerve transfers have emerged as a key reconstructive technique, particularly valuable in cases where primary repair or grafting is unfeasible or delayed. This retrospective study analysed functional outcomes following nerve transfers in 37 patients with brachial plexus injury. Motor recovery was assessed using the Medical Research Council scale. Patients were stratified by age, timing of surgery, injury severity, and type of nerve transfer performed. The majority of our cohort consisted of male adults, predominantly injured in motorcycle accidents, while pediatric cases were mostly due to obstetrical trauma. For shoulder reanimation, all patients received spinal accessory to suprascapular nerve transfer, with a subset also undergoing medial triceps branch of the radial nerve to axillary nerve transfer. These techniques resulted in 85.3% of patients achieving shoulder function recovery with M3 or M4 muscle strength, with combined procedures leading exclusively to M3 or M4 muscle strength. For elbow flexion restoration, surgical approaches included intercostal to musculocutaneous nerve transfer, ulnar and median fascicles to musculocutaneous nerve transfer, contralateral C7 to musculocutaneous nerve transfer with ulnar graft, and spinal accessory to musculocutaneous nerve transfer with sural nerve graft. Root grafting procedures using sural nerve grafts or nerve conduits were employed in three pediatric patients. Overall, 84.38% of patients achieved elbow flexion recovery with M3 or M4 muscle strength. These findings reinforce the utility of nerve transfers as a cornerstone in the surgical management of brachial plexus injury.

## INTRODUCTION

Brachial plexus lesions are rare but severely disabling, causing substantial loss of motor and sensory function in the upper limb. These injuries are often caused by high-velocity trauma, including motor vehicle accidents and especially motorcycle crashes [1-3]. Another cause for brachial plexus injury is birth trauma, which can occur in 1 out of 1000 births [4,5]. Affected individuals typically experience paralysis, sensory loss, and intense neuropathic pain, all of which have a profound impact on their quality of life [3,6-9]. Historically, treating brachial plexus injuries has been challenging, as traditional methods like tendon transfers and muscle grafts often lead to less-than-ideal functional outcomes. This is due to the long distances required for nerve regeneration and the associated muscle atrophy. Although microsurgical techniques have advanced, functional recovery remains limited, especially in patients with complete brachial plexus injuries, where nerve regeneration involves greater distances [1,10-13].

Nerve transfers have emerged as a transformative approach in brachial plexus reconstruction, addressing the limitations of traditional methods. By converting proximal injuries into distal lesions closer to target muscles, nerve transfers enhance the probability of prompt reinnervation and functional recovery. This technique avoids scarred tissue planes, reduces the need for grafting, and prevents the degeneration of motor endplates associated with delayed reinnervation. Building on the pioneering work of Oberlin *et al*., nerve transfers have become fundamental to modern peripheral nerve surgery, enabling more precise and efficient repair of these complex injuries [14-17].

### Brachial plexus anatomy

After the spinal nerves emerge from the spinal cord, the roots divide into a posterior and an anterior ramus. The anterior divisions of C5 to T1 spinal nerves converge to form the brachial plexus. As the roots pass through the supraclavicular fossa, they form three trunks. The superior trunk is formed by the roots of C5 and C6 nerve roots, the middle trunk rises from the C7 nerve root, and the inferior trunk is formed from the C8 and T1 nerve roots. After the nerve trunks travel along the infraclavicular space, they split to form two divisions, an anterior and a posterior division. The three posterior divisions unite and form the posterior cord, travel posteriorly related to the axillary artery, and form the axillary nerve, the radial nerve, the thoracodorsal nerve, and the superior and inferior subscapular nerves. The anterior divisions from the superior and middle trunk join to form the lateral cord, giving birth to the musculocutaneous nerve, the lateral fascicles of the median nerve, and the lateral pectoral nerve. The medial cord continues the anterior division of the inferior trunk and then forms the ulnar nerve, the medial fascicles of the median nerve, the medial brachial and antebrachial nerves, and the medial pectoral nerve [18-20].

### Classification of brachial plexus injuries

**Figure 1:**
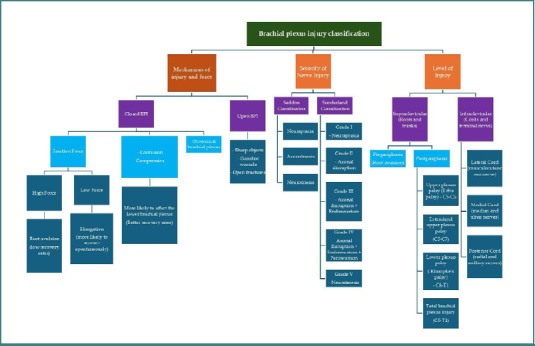
Classification of brachial plexus injuries [3,7,21,22]

Brachial plexus injuries can be classified based on the mechanism of injury and injury force, the severity of nerve injury, and injury level. A detailed classification is presented in Figure 1.

### Management of brachial plexus injuries

The timing of the surgery is one of the most important decisions in brachial plexus injuries, and the treatment plan is decided based on the mechanism of injury and the time elapsed from the moment of injury. If the injury occurred after penetration with a sharp object, then acute exploration and direct coaptation of the injured nerves is done if possible. In cases of open wounds that occurred following blunt force trauma, nerve repair should be delayed until after 3-4 weeks, so that the injury zone has fully evolved [23,24]. In closed brachial plexus injuries, the surgery should be delayed for at least 3 months to allow for spontaneous nerve regeneration. Following the 3 months, another clinical and paraclinical examination should be performed to assess if any progress has been made. If there are signs of nerve regeneration, the conservative treatment is maintained, and further clinical examinations are done periodically. If there are no signs of recovery, surgery should be performed between 3 and 6 months following the injury [23,24].

Direct nerve repair is the preferred treatment option in open brachial plexus injuries when immediate exploration reveals that the nerve ends can be approximated without tension [25]. In postganglionic lesions resulting in a nerve gap unsuitable for direct repair, nerve grafts are used after the excision of the injured area and the preparation of the nerve stumps. In preganglionic injuries, where nerve roots are avulsed from the spine, nerve grafting is no longer possible. In recent years, when nerve transfers have gained more and more popularity, root exploration with nerve grafting has become less utilized, although there are studies show that root exploration and grafting together with nerve transfers can yield better results [23,26].

Nerve transfers can be employed when the nerve roots are ruptured from the spinal cord, when nerve grafting is impossible, or as a first choice to decrease the distance that axons travel towards the target muscle, thereby achieving a faster reinnervation. In recent years, nerve transfers have gained more and more usage because of their advantages, such as bypassing the injury zone, faster recovery, decreasing the potential destruction of the motor end plates, and the ability to use them in cases of delayed presentation [23,25,26].

### Indication of nerve transfers

Nerve transfers offer an alternative approach when traditional reconstructive techniques, such as nerve grafting or tendon transfer, may not achieve optimal functional outcomes. A key indication for nerve transfer is the lack of a proximal nerve stump, which makes direct repair or grafting challenging or impossible. Additionally, nerve transfers prove especially useful when treatment is delayed, as they can still facilitate functional recovery up to 8 to 10 months after the injury, shortening the time needed for reinnervation of the target muscles [15,27,28].

Recent advancements have extended the use of nerve transfer in treating birth-related brachial plexus injuries, providing a promising method for restoring both motor and sensory function in these patients. While nerve transfers were generally used for lesions of the brachial plexus that were beyond repair, their applications have expanded due to the observation of suboptimal functional outcomes even after direct repair. As a result, nerve transfer has become a more viable option for patients with these types of injuries [9,29-33].

Nerve transfers are valuable when the remaining tissue cannot provide an optimal environment for nerve grafting or regeneration, particularly in complex upper limb injuries, or when primary repair or other options, such as tendon transfer, are not viable or would result in poor outcomes. Unlike tendon transfer, which moves expendable musculotendinous units, nerve transfer preserves the natural musculotendinous vectors, potentially leading to better functional results. However, nerve transfer cannot be postponed indefinitely, and in some instances, tendon transfer may offer superior results [1,34].

The most frequently observed pattern of brachial plexus injury is superior trunk lesions (C5-C6), especially in the adult population [1,35,36]. This injury damages the suprascapular, axillary, and musculocutaneous nerves, affecting shoulder abduction and elbow flexion. Damage to the C7 root can add a deficit in wrist and elbow extension due to radial nerve lesion [1,3,37].

Total brachial plexus injuries entail lesions to all the roots of the brachial plexus, leading to complete paralysis of the shoulder, elbow, wrist, and hand. In contrast to injuries in the upper brachial plexus, pan-brachial plexus damage leaves limited donor nerves for nerve transfers, with most viable options coming from extraplexal sources. The prognosis for total brachial plexus injuries is generally poor, particularly for restoration of hand mobility, compared to the more favorable outcomes observed in upper plexus injuries [1,3,38].

Elbow flexion and shoulder function have a crucial role in the upper limb autonomy, so, regardless of the level of injury, restoring these functions should be the primary focus of surgical repair [1,9].

The primary goal of our study was to assess the surgical outcomes of nerve transfers for shoulder function and elbow flexion in patients with brachial plexus injuries. Secondary objectives included comparing results between total brachial plexus and upper brachial plexus injuries, between the obstetric and adult populations with brachial plexus injuries, and our results with the existing literature.

## MATERIAL AND METHODS

We conducted a retrospective study of cases admitted over 10 years, from January 2015 to December 2024, in the plastic surgery departments of Zetta Hospital in Bucharest, the Clinical Emergency Hospital of Bucharest, and the Emergency Clinical Hospital for Children M.S. Curie. The study focused on patients with brachial plexus injuries, out of whom we selected only those who underwent surgical treatment involving nerve transfers. Patients lacking complete medical records and patients who underwent other surgical procedures for brachial plexus injury were excluded from the study.

At the initial consultation, a detailed medical history was taken. A thorough general and local clinical examination focused on identifying specific injuries and functional impairments. The patient was informed about the available treatment options and the associated benefits and risks of each approach. Based on this assessment, a customized surgical plan was developed. On admission to the hospital, all patients consented to their medical data being used for future research purposes.

The collected variables in our study included the patient’s name, age, sex, site of the injury, mechanism of injury, time elapsed from the accident to the surgical intervention, follow-up period, type of nerve transfer performed, and motor recovery outcome. All data were collected and processed using Microsoft Excel, version 16.66.1.

Based on the principle that elbow flexion and shoulder function are the most important and should be the first to reanimate, our patients benefited from one or more of the following nerve transfers:

### Spinal accessory to suprascapular nerve transfer for shoulder function (Figure 2)

**Figure 2:**
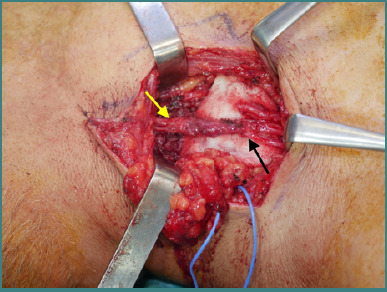
Spinal accessory to suprascapular nerve transfer. The yellow arrow points towards the spinal accessory nerve, and the black arrow points towards the suprascapular nerve.

The surgery was performed under general anesthesia, with the patient in a supine position. An anterior approach was used to make an incision in the supraclavicular fossa. Following the skin incision, a meticulous dissection was carried out until the spinal accessory nerve was identified along the superolateral edge of the trapezius muscle and dissected as distally as possible.

The suprascapular nerve was located at the scapular notch and was transposed medially for easier coaptation. A nerve stimulator was used to facilitate nerve identification and to confirm that the suprascapular nerve was non-responsive to electrical stimulation. After identification, the nerves were transected. Once the nerve stumps were carefully prepared, an end-to-end anastomosis was performed between the spinal accessory and suprascapular nerve using a 9.0 Prolene suture.

### Medial triceps nerve to axillary nerve transfer for shoulder function (Figure 3)

**Figure 3:**
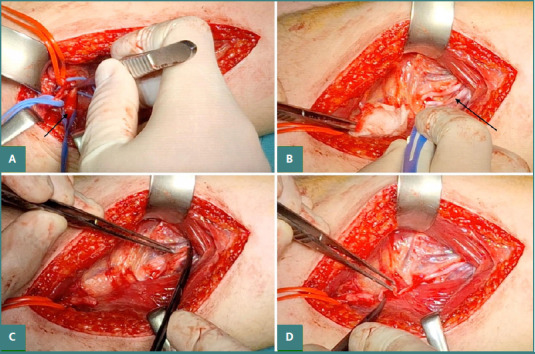
Radial nerve fascicle to axillary nerve transfer. A, Axillary nerve identification (black arrow); B, Radial nerve fascicle identification with nerve stimulator (black arrow pointing towards the chosen fascicle for transfer); C, Cutting of the radial nerve fascicle; D, Preparation of axillary and radial fascicle nerve stamps for anastomosis.

The surgery was performed under general anesthesia. An incision was made on the posterior aspect of the arm from the middle third of the arm towards the posterior border of the deltoid muscle. After the skin incision and careful dissection, the axillary nerve was identified in the quadrangular space and released as proximally as possible. The radial nerve was identified between the lateral and the long heads of the triceps muscle. A nerve stimulator was used to facilitate nerve identification and selection of the nerve for the medial head of the triceps and to confirm that the axillary nerve was non-responsive to electrical stimulation. After identification, the nerves were transected and prepared for coaptation. An end-to-end anastomosis was performed using a 9.0 Prolene suture.

### Branches from the ulnar and median nerves to the musculocutaneous nerve transfer for elbow flexion

The technique was first described by Oberlin in 1994, proposing using a branch from the ulnar nerve to the musculocutaneous nerve to recover elbow flexion [17]. Humphreys and Mackinon added the use of median fascicle to enhance the result [39]. With the patient in a supine position, under general anesthesia, a skin incision was made on the medial aspect of the arm; after careful dissection the ulnar, median and musculocutaneous nerves were identified, an intraneural dissection was performed on the ulnar and median nerves and, using a nerve stimulator, the fascicles for the flexor carpi ulnaris muscle from the ulnar nerve and the fascicle for the flexor carpi radialis muscle from the median nerve were isolated. The two branches were transected after identifying the motor branches for the biceps brachialis muscles from the musculocutaneous nerve and ensuring that they were non-responsive to electrical stimulation. An end-to-end anastomosis was performed between the ulnar fascicle and the brachialis branch and between the median fascicle and the biceps brachialis branch with a 9.0 Prolene suture.

### Intercostal nerves to musculocutaneous nerve transfer for elbow flexion (Figure 4)

**Figure 4:**
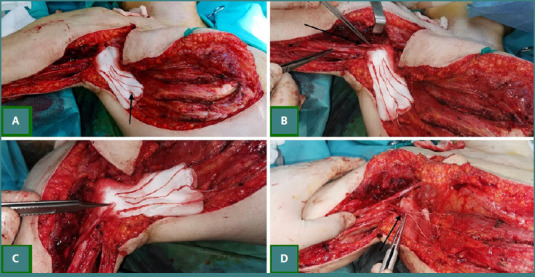
Intercostal nerves to the musculocutaneous nerve transfer. A, Intercostal nerve harvested for transfer (black arrow); B, Musculocutaneous nerve identification (black arrow); C, Preparation of musculocutaneous and intercostal nerve stamps for anastomosis; D, Anastomosis between musculocutaneous and intercostal nerves.

The intercostal nerves were used in patients with complete brachial plexus injuries, when the ulnar and median nerves were not viable. Under general anesthesia, the patient was placed supine, and a curved 'L'-shaped incision was made from the anterior axillary fold along the lateral thoracic wall to the inframammary crease. The pectoralis major was elevated, and the rib periosteum incised to allow subperiosteal dissection and exposure of the intercostal vascular pedicle. With a nerve stimulator, the motor branches of three consecutive intercostal nerves were identified, divided, and mobilized toward the anterior axillary line. The musculocutaneous nerve was identified in the upper third of the arm and dissected towards the branching to the biceps and brachialis muscles, and after checking that the muscles were non-responsive to electrical stimulation, the nerve was transected. The three intercostal nerves were reflected towards the musculocutaneous nerve stump, and an end-to-end anastomosis was performed with a 9.0 Prolene suture.

### Contralateral C7 to the musculocutaneous nerve transfer for elbow flexion

**Figure 5:**
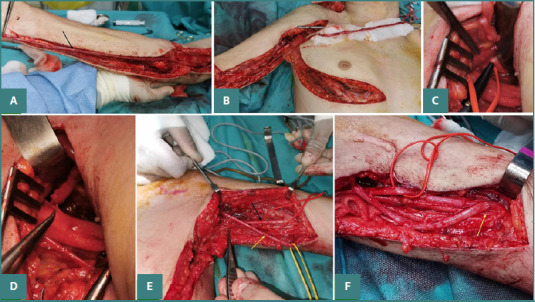
Contralateral C7 to median nerve transfer A, Ulnar nerve harvested for transfer (black arrow); B, Ulnar nerve reflected towards contralateral C7 through a subcutaneous tunnel C; C, C7 root identification; D, Anastomosis between Ulnar nerve and contralateral C7; E, Identification of ulnar (yellow arrow) and median (black arrow) nerves; F, Anastomosis between Ulnar and Median nerves

The contralateral C7 to musculocutaneous nerve transfer was first performed by Gu *et al*. in 1992 [40]. Since then, various techniques have been described. In our practice, the contralateral C7 to musculocutaneous nerve transfer is done in two stages with a vascularized ulnar nerve graft. The surgery is performed under general anesthesia, with the patient in supine position, an incision is made from the ulnar aspect of the volar side of the forearm on the affected side from the wrist going all the way to the axilla, with multiple triangular flaps (Z-plasty) created at the cubital fossa and the axilla, meticulous dissection is performed at the ulnar side of the wrist and the ulnar vascular pedicle is identified, the nerve is isolated and dissected, an electrical stimulation is performed to make sure that there is no response. The nerve is transected and utilized as a vascularized nerve graft, which is reflected at the proximal third of the arm. Another incision is made at the opposite side at the supraclavicular fossa, and a careful dissection is performed to access the brachial plexus roots. With the help of a nerve stimulator, the C7 root is identified, and a partial transection is performed on the C7 root. A subcutaneous tunnel is created above the pectoral plane, and the ulnar nerve graft is passed through the tunnel towards the contralateral C7 root. An end-to-end anastomosis is performed between the ulnar nerve and the C7 root with a 9.0 Prolene suture. Once we observe objective signs of ulnar graft reinnervation in the axilla, typically between 7 and 9 months post-transfer, an incision is made at the site of the old scar at the proximal third of the arm. After dissection, the ulnar nerve graft and the musculocutaneous nerves are identified and transected. After preparing the nerve stumps, an end-to-end anastomosis is performed between the ulnar graft and the musculocutaneous nerve with a 9.0 Prolene suture. Figure 5 shows the contralateral C7 to the median nerve transfer, but the same principles and steps applies to the contralateral C7 to the musculocutaneous nerve transfer.

### Spinal accessory to musculocutaneous nerve transfer for elbow flexion

The spinal accessory nerve can be used as a nerve donor for elbow reanimation in complete brachial plexus injuries if the other possible donors are unavailable. In our cases, the spinal accessory was chosen in patients with previous surgeries on the thoracic wall, where intercostal nerves were not an option.

With the patient in the supine position, under general anesthesia, a skin incision was made in the supraclavicular fossa, going all the way to the proximal third of the medial aspect of the arm. Dissection was performed to identify the spinal accessory nerve as previously described. The musculocutaneous nerve was discovered between the brachialis and the biceps muscles and stimulated with a nerve stimulator to ensure the nerve was unresponsive to electrical stimulation. The accessory and musculocutaneous nerves were transected, the nerve stamps were prepared for coaptation, and a sural nerve graft was harvested. The nerve graft was then interposed between the nerve stumps of the spinal accessory and musculocutaneous nerves, and end-to-end anastomosis was performed with 9.0 Prolene suture.

The Medical Research Council (MRC) scale was utilized to evaluate the motor function recovery of both shoulder function and elbow flexion (Table 1).

**Table 1:** MRC scale for muscle strength [41]

Grade	Muscle strength
M0	No muscular contraction
M1	Signs of muscular contraction and fasciculation, no movement
M2	Movement with gravity removed
M3	Movement against gravity
M4	Movement against resistance
M5	Normal muscle strength

## RESULTS

We included in our study a total of 37 patients who suffered brachial plexus injuries and underwent nerve transfer surgeries from 2015 to 2024. The majority of our patients were men (34 out of 37). The three female patients in our study were children who suffered obstetrical injuries. The age distribution was between 1 and 77 years old, with a mean age of 26.8. We had nine pediatric patients and 28 adults.

**Figure 6:**
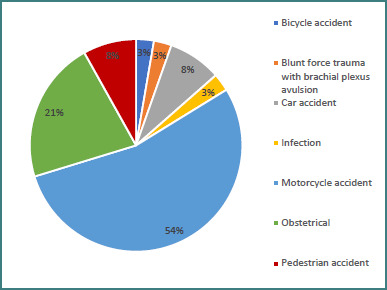
Mechanism of injury to the brachial plexus

Regarding the mechanism of injury, we observed that most of our adult population sustained their injury in road accidents (27 out of 28), with more than 50% of our total population representing motorcycle accidents (54%). From our pediatric population, 8 out of 9 children experienced obstetrical injuries, and one of them sustained their injury due to polyneuropathy following an infection (Figure 6).

Concerning the level of injury, from a total of 37 patients, 18 patients suffered a complete brachial plexus injury and 19 a partial brachial plexus injury (18 C5-C6 roots and 1 C5-C6-C7). From our total population, 24 patients sustained the injury on the left upper limb (64,86 %) and 13 on the right upper limb.

**Figure 7:**
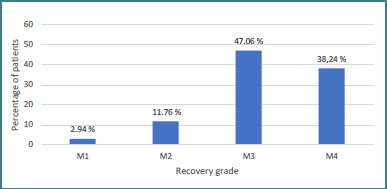
Global shoulder function recovery grade

**Figure 8:**
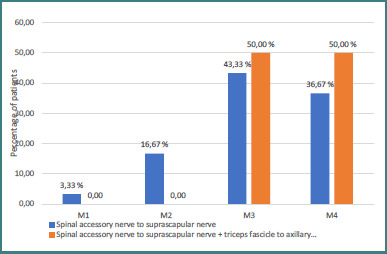
Global shoulder function recovery in relation to the surgical procedure used

Of our 37 patients, 34 underwent nerve transfer surgery for shoulder reanimation. All of them received the spinal accessory to suprascapular nerve transfer. In addition, four of them also benefited from the medial fascicle of the radial nerve, which innervates the triceps, to axillary nerve transfer. According to the MRC scale, 85.3% of patients achieved a shoulder function recovery of M3 or higher following nerve transfer surgery. Specifically, 47.06% reached M3 strength, while 38.24% attained M4. Only a small proportion demonstrated lower recovery grades, with 11.76% reaching M2 and 2.94% remaining at M1 (Figure 7). In cases where a combined procedure (spinal accessory nerve to suprascapular nerve transfer and the medial triceps nerve to axillary nerve transfer) was performed, all patients obtained a result of M3 or M4 (Figure 8).

**Figure 9:**
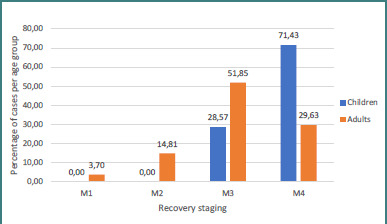
Shoulder function recovery in children versus adults

**Figure 10:**
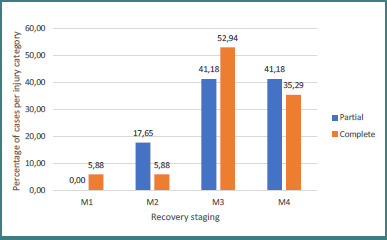
Shoulder function recovery in partial versus complete injuries

Regarding the results based on age groups, we observed that all the children with shoulder function procedures obtained a result of M3 or better compared with 81.48% in our adult population, while 71.43% of children obtained an M4 result, compared with 29.63% of the adults (Figure 9). Furthermore, results in our study showed that 88.23% of the patients with complete injuries gained a result of M3 or better compared with 82.36% of the partial brachial plexus injuries, but 41.18% of them gained a result of M4 or better compared with 35.29% from the complete brachial plexus injury population (Figure 10).

**Figure 11:**
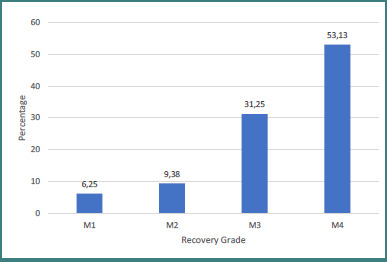
Global elbow flexion recovery grade

Procedures to restore elbow flexion were done in 32 out of 37 patients. Of these, 29 underwent nerve transfers, and three pediatric patients also received C5 root grafting to the upper trunk (two of them with sural nerve graft and one with nerve conduit) and C6 root grafting to the middle trunk with sural nerve graft. The 29 nerve transfer procedures were distributed as follows: 13 intercostal to musculocutaneous nerve transfers, 11 ulnar and median fascicles to musculocutaneous nerve transfers, two contralateral C7 to musculocutaneous nerve transfers with ulnar nerve graft, two spinal accessory to musculocutaneous nerve transfers with sural nerve graft and one C7 fascicles to distal C5 and C8 fascicles to distal C6 nerve transfer (in pediatric patient). Among our patients, 84.38% obtained a result of M3 or better according to the MRC scale (Figure 11).

**Figure 12:**
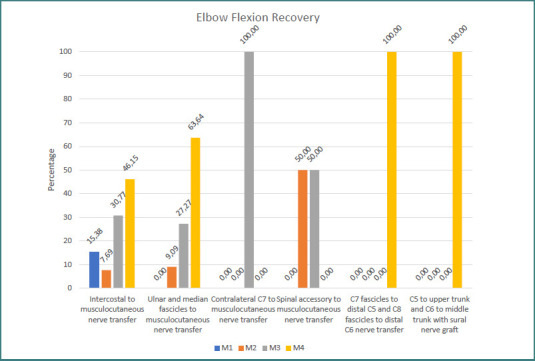
Global elbow flexion recovery grade in relation to the surgical procedure used

Recovery of elbow flexion to M3 or M4 was achieved in 76.9% of patients following intercostal-to-musculocutaneous nerve transfers and in 90.9% of those who underwent ulnar and median fascicle transfers to the musculocutaneous nerve. All patients (100%) receiving contralateral C7-to-musculocutaneous transfers with a vascularized ulnar graft attained M3–M4 recovery, whereas only 50% of the patients with transfer of the spinal accessory to the musculocutaneous nerve reached this level. 100% of the patients with transfer of C7 fascicles to distal C5 and transfer of C8 fascicles to distal C6 nerve, and 100% of the C5 root grafting to the upper trunk and C6 root grafting to the middle trunk with sural nerve graft group achieved M3–M4 recovery rates (Figure 12).

**Figure 13:**
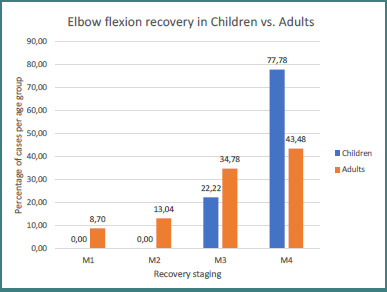
Elbow flexion recovery in children versus adults

Analysis by age group showed that all pediatric patients achieved M3 or better elbow flexion, compared with 78.3% of adults; 77.8% of children reached M4 versus 43.5% of the adult cohort (Figure 13).

**Figure 14:**
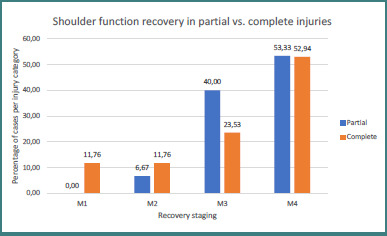
Elbow flexion recovery in partial vs. complete injuries

Concerning the results based on partial vs complete brachial plexus injuries, we found out that the group with partial brachial plexus injuries had a better result compared with the complete brachial plexus injury group (93.33% vs 76.47% of M3 and M4 recovery stages) (Figure 14).

In our cohort of 37 patients, the time between the accident and surgery ranged from 2 to 24 months, with an average of 8.03 months. The mean follow-up time was 3.32 years, ranging from 1 to 9 years.

## DISCUSSION

Brachial plexus damage generally occurs in young individuals, more frequently in men, with high-speed road accidents being the leading cause. Avulsion injuries represent 60% of these injuries, which have less favorable prognoses compared to open sharp trauma [12, 32]. These types of injuries frequently lead to lesions of the supraclavicular plexus, with around 50% involving the C5, C6, and occasionally C7 roots, leading to compromised functions of the shoulder and elbow [42,43]. Partial brachial plexus injuries, particularly ones involving the upper root, tend to show better functional recovery, with elbow flexion and shoulder function generally regarded as the primary focus in brachial plexus treatment plans 31,44,45]. In our study, 73% of our patients suffered closed avulsion injuries from road accidents, most of them involving a motorcycle. 51% of our patients suffered C5-C6/C7 injuries.

Nerve surgery has greatly benefited from advancements in microsurgery and anatomical studies of nerve fascicles, leading to improved surgical techniques for nerve coaptation. However, achieving functional reanimation after brachial plexus injuries remains challenging, particularly in proximal lesions [9,46]. Several factors are involved in less favorable outcomes, such as length of nerve defect, surgical technique, associated injuries, age, but time and distance are the most important for brachial plexus injuries [9,47].

The regeneration rate of nerves following an injury is about 1 to 3 mm daily. This leads to a narrow time frame for reconstruction to achieve optimal results. Studies recommend that the target reinnervation should be achieved between 12 and 18 months. Otherwise, the motor end plates will suffer irreversible denervation. Muscles affected by this process are primarily located farthest from the level of injury. This translates into progressively poorer results for the distal muscles compared to those closer to the site of nerve reconstruction [1,46,48-50].

Nerve transfers were initially adopted for traction injuries of the brachial plexus that resulted in the avulsion of the roots, rendering them unusable for repair. After observing the benefits of achieving good reanimation, nerve transfers began to gain popularity even in cases of brachial plexus lesions without root avulsions [9,51].

This shift from traditional reconstruction techniques to nerve transfers is supported by a series of benefits. Firstly, nerve transfers offer another site of neurotization. This comes in handy especially when direct coaptation is not an option, or when we anticipate a poor functional outcome primarily because of the distance from the injury to the targeted muscle or the time elapsed before the patient is addressed. Combining nerve transfers with direct repair or root nerve grafting can lead to a better outcome than nerve grafting alone. Another advantage of nerve transfers is that they are performed near the recipient muscles, leading to a faster restoration of function [1,51-55].

Regarding shoulder function recovery after nerve transfers, studies show that an optimal result of MRC grade 3 or better was achieved in 60 to 100% of cases [9,17,56-59]. The ones reporting close to 100% of cases recovering a good functional outcome generally have a small sample of patients. A systematic review by Yang *et al*., including 104 patients who benefited from nerve transfers for shoulder function, reported an M3 or better result in 82% of the patients [42]. Our case series included 34 patients who underwent nerve transfer surgery for shoulder reanimation. Of the 34 patients, 30 underwent spinal accessory to suprascapular nerve transfer alone, and four benefited from dual nerve transfer (spinal accessory to suprascapular nerve transfer and medial triceps to axillary nerve transfer). The mean follow-up period was 3.32 years. 85.3% of our patients obtained a result of M3 or better according to the MRC scale, and 80% of those with single nerve transfer obtained an M3 or better result. Our results are comparable to those found in the literature. The patients who benefited from dual nerve transfer obtained a result better than M3 in 100% of our cases. This is in accordance with the findings in the literature that show that dual nerve transfers can achieve better results than single nerve transfers for shoulder reanimation, although our sample population was small [11, 57-63].

In our study population, 29 out of our 37 patients underwent nerve transfers to restore elbow flexion. The nerve transfer procedures were distributed as follows: 13 intercostal to musculocutaneous nerve transfers, 11 ulnar and median fascicles to musculocutaneous nerve transfers, two contralateral C7 to musculocutaneous nerve transfers with ulnar nerve graft, two spinal accessory to musculocutaneous nerve transfers with sural nerve graft and one C7 fascicles to distal C5 and C8 fascicles to distal C6 nerve transfer (in a pediatric patient). The mean follow-up period was 3.32 years. 84.38% of our patients obtained a result of M3 or better according to the MRC scale. Other studies showed results of M3 or better, ranging from 73.4 % to 100%, consistent with our findings [58,64-67]. A meta-analysis from Texakalidis *et al*., including 350 patients with nerve transfers for elbow flexion, showed that 77.7 % obtained an M3 or better result [68].

Most of our patients (24 out of 32 who underwent surgery to restore elbow flexion) benefited from the intercostal to musculocutaneous nerve transfer or ulnar and median fascicles to the musculocutaneous nerve. 76.92% of the patients from the intercostal to musculocutaneous nerve transfer groups achieved M3 or better. Other studies show recovery rates of M3 or better between 57% and 100% of cases, with the mean around 72% [9,69-73]. 90.91% of the patients from the ulnar and median fascicles to the musculocutaneous nerve transfer group obtained a good or excellent result (M3 or better). Studies from the literature support our findings with results of M3 ranging between 75% and 100% of the cases [74-77]. Regarding results comparing the two techniques, studies show better results of the double nerve transfer (ulnar and median fascicles) than the intercostal to musculocutaneous nerve transfer, which is also supported by our findings [78,79].

Nerve transfers are the main surgical technique for managing brachial plexus lesions in the adult population. In obstetrical brachial plexus injuries, on the other hand, the general management, if nerve regeneration is not observed following conservative treatment, is excision of the neuroma and reconstruction with nerve grafts. Lately, the use of nerve transfers has been implemented as the main treatment for obstetrical brachial plexus as well, with very good results [4,5,80-82]. In our study, we included nine pediatric patients, eight obstetrical brachial plexus injuries, and one patient injured due to polyneuropathy following an infection. We performed seven nerve transfers for shoulder reanimation (spinal accessory to suprascapular nerve), six of them for obstetrical brachial plexus, with 100% of our patients recovering an M3 or better function, and five of them achieved an M4 result. For elbow flexion reanimation, we performed three root grafts and six nerve transfers (four times the fascicles from ulnar and median nerves to musculocutaneous nerve transfer, one spinal accessory to musculocutaneous nerve transfer, and one C7 fascicle to distal C5 and C8 fascicles to distal C6 nerve transfer). All patients obtained an M3 or better result; seven had an M4 result. The results in our pediatric population are significantly better than those in our adult population, which is expected because of the greater capacity of nerve regeneration and the smaller distance for the axons to travel, due to the dimension of the superior extremity [5, 83].

Our results comparing elbow function after nerve transfers in partial versus complete brachial plexus injuries show that better results are obtained following partial brachial injuries (93.33% vs 76.47% of M3 or better recovery). This is consistent with other clinical studies showing similar results. Ayhan *et al*. observed that 91.9% of the patients with partial brachial plexus injuries obtained a result of M3 or better on the MRC scale compared with 74.3% following complete brachial plexus injuries [64]. Another study conducted in 2014 by Xiao *et al*. showed that, from the partial brachial plexus injury group, 86.7% obtained satisfactory elbow recovery, whereas only 66.7% of the patients from the complete brachial plexus injury group obtained similar results [69]. These results could be explained by the fact that brachial plexus injuries require extraplexal nerve transfers, translating to a longer path that axons must travel before reaching their target muscles. Another setback is that extraplexal nerve transfers need corticalisation for the reinnervated muscle to function correctly. This translates to longer rehabilitation periods [84].

Regarding shoulder function after nerve transfers in complete versus partial brachial plexus injuries, we obtained similar results between the two groups (82.36% vs 88.23% of M3 or better MRC scale results). These can be explained by the fact that the spinal accessory to suprascapular nerve transfer is the main nerve transfer for shoulder function, regardless of the level of injury, so we can expect similar results. An amendment can be made that in partial brachial plexus injuries where the radial nerve is intact, we can add a second nerve transfer for shoulder function (medial triceps nerve to axillary nerve transfer) that can yield better results than spinal accessory to suprascapular nerve transfer alone.

## CONCLUSION

Nerve transfers represent a reliable and effective surgical strategy for restoring shoulder and elbow function in patients with brachial plexus injuries, particularly when primary repair is not feasible. This multicenter retrospective analysis highlights favorable functional outcomes, with over 84% of patients regaining meaningful motor recovery in both shoulder and elbow movements. Pediatric patients and those with partial injuries demonstrated notably better results, emphasizing the importance of early diagnosis and individualized surgical planning. These findings reinforce the utility of nerve transfers as a cornerstone in the surgical management of brachial plexus injuries, warranting continued refinement and adoption of tailored approaches to optimize patient outcomes.

## Data Availability

Further data are available from the corresponding author upon reasonable request.

## References

[1] Colbert SH, Mackinnon SE (2008). Nerve transfers for brachial plexus reconstruction. Hand Clin.

[2] Midha R Epidemiology of brachial plexus injuries in a multitrauma population. Neurosurgery1997;.

[3] Limthongthang R, Bachoura A, Songcharoen P, Osterman AL (2013). Adult brachial plexus injury: evaluation and management. Orthop Clin North Am.

[4] Pondaag W, Malessy MJ, van Dijk JG, Thomeer RT (2004). Natural history of obstetric brachial plexus palsy: a systematic review. Dev Med Child Neurol.

[5] Davidge KM, Clarke HM, Borschel GH (2016). Nerve Transfers in Birth Related Brachial Plexus Injuries: Where Do We Stand?. Hand Clin.

[6] Kim DH, Midha R, Murovic JA, Spinner RJ, Teil R (2007). Kline and Hudson’s nerve injuries: operative results for major nerve injuries, entrapments and tumors.

[7] Mackinnon SE (1989). New directions in peripheral nerve surgery. Ann Plast Surg.

[8] Terzis JK, Kostas I, Soucacos PN (2006). Restoration of shoulder function with nerve transfers in traumatic brachial plexus palsy patients. Microsurgery.

[9] Sulaiman OA, Kim DD, Burkett C, Kline DG (2009). Nerve transfer surgery for adult brachial plexus injury: a 10-year experience at Louisiana State University. Neurosurgery.

[10] Yeoman P, Seddon H (1961). Brachial plexus injuries: treatment of the flail arm. J Bone Joint Surg Br.

[11] Leechavengvongs S, Witoonchart K, Uerpairojkit C, Thuvasethakul P (2003). Nerve transfer to deltoid muscle using the nerve to the long head of the triceps, part II: a report of 7 cases. J Hand Surg Am.

[12] Dubuisson AS, Kline DG (2002). Brachial plexus injury: a survey of 100 consecutive cases from a single service. Neurosurgery.

[13] Midha R (2006). Emerging techniques for nerve repair: nerve transfers and nerve guidance tubes. Clin Neurosurg.

[14] Brown JM, Mackinnon SE (2008). Nerve transfers in the forearm and hand. Hand Clin.

[15] Tung TH (2014). Nerve transfers. Clin Plast Surg.

[16] Zhang D, Varadharajan V, Bhardwaj P, Venkatramani H, Sabapathy SR (2022). Considerations in the Selection of Donor Nerves for Nerve Transfer for Reanimation of Elbow and Shoulder in Traumatic Brachial Plexus Injuries. J Hand Surg Asian Pac Vol.

[17] Oberlin C, Beal D, Leechavengvongs S, Salon A, Dauge MC, Sarcy JJ (1994). Nerve transfer to biceps muscle using a part of ulnar nerve for C5–C6 avulsion of the brachial plexus: Anatomical study and report of four cases. J Hand Surg Am.

[18] Li H, Chen J, Wang J, Zhang T, Chen Z (2023). Review of rehabilitation protocols for brachial plexus injury. Front Neurol.

[19] Rubin DI (2020). Brachial and lumbosacral plexopathies: A review. Clin Neurophysiol Pract.

[20] Singh DK, Kumar N, Bhayana A, Altamash M, Sharma A, Agarwal A (2023). A pentavalent approach for the evaluation of traumatic brachial plexopathy on MRI: correlation of macropattern and micropattern. Br J Radiol.

[21] Thatte MR, Babhulkar S, Hiremath A (2013). Brachial plexus injury in adults: Diagnosis and surgical treatment strategies. Ann Indian Acad Neurol.

[22] Sunderland S (1978). Nerves and Nerve Injuries.

[23] Noland SS, Bishop AT, Spinner RJ, Shin AY (2019). Adult Traumatic Brachial Plexus Injuries. J Am Acad Orthop Surg.

[24] Mackinnon SE, Dellon AL, Mackinnon SE, Dellon AL (1988). Brachial plexus injuries. Surgery of the Peripheral Nerve.

[25] Tung TH, Moore AM, Mackinnon SE (2015). Brachial plexus injuries. Nerve Surgery.

[26] Sinha S, Khani M, Mansoori N, Midha R (2016). Adult brachial plexus injuries: Surgical strategies and approaches. Neurol India.

[27] Tung TH, Liu DZ, Mackinnon SE (2009). Nerve transfer for elbow flexion in radiation-induced brachial plexopathy: a case report. Hand (N Y).

[28] Novak CB, Mackinnon SE (2004). Treatment of a proximal accessory nerve injury with nerve transfer. Laryngoscope.

[29] Kozin SH (2008). Nerve transfers in brachial plexus birth palsies: indications, techniques, and outcomes. Hand Clin.

[30] Kline DG, Tiel RL (2005). Direct plexus repair by grafts supplemented by nerve transfers. Hand Clin.

[31] Songcharoen P, Wongtrakul S, Spinner RJ (2005). Brachial plexus injuries in the adult. nerve transfers: the Siriraj Hospital experience Hand Clin.

[32] Terzis JK, Kostopoulos VK (2007). The surgical treatment of brachial plexus injuries in adults. Plast Reconstr Surg.

[33] Weber RV, MacKinnon SE (2004). Nerve transfers in the upper extremity. J Am Soc Surg Hand.

[34] Guelinckx PJ, Carlson BM, Faulkner JA (1992). Morphologic characteristics of muscles grafted in rabbits with neurovascular repair. J Reconstr Microsurg.

[35] Sakellariou VI, Badilas NK, Mazis GA, Stavropoulos NA, Kotoulas HK, Kyriakopoulos S (2014). Brachial plexus injuries in adults: evaluation and diagnostic approach. ISRN Orthop.

[36] Reichert P, Kiełbowicz Z, Dzięgiel P, Puła B, Wrzosek M, Bocheńska A (2016). Effect of Collateral Sprouting on Donor Nerve Function After Nerve Coaptation: A Study of the Brachial Plexus. Med Sci Monit.

[37] Tsai YJ, Hsiao CK, Su FC, Tu YK (2022). Clinical Assessment of Functional Recovery Following Nerve Transfer for Traumatic Brachial Plexus Injuries. Int J Environ Res Public Health.

[38] Widodo W, Dilogo IH, Kamal AF, Antarianto RD, Wuyung PE, Siregar NC (2024). Functional outcome and histologic analysis of late onset total type brachial plexus injury treated with intercostal nerve transfer to median nerve with local umbilical cord-derived mesenchymal stem cells or secretome injection: a double-blinded, randomized control study. Eur J Orthop Surg Traumatol.

[39] Humphreys DB, Mackinnon SE (2002). Nerve transfers. Oper Tech Plast Reconstr Surg.

[40] Gu YD, Zhang GM, Chen DS, Yan JG, Cheng XM, Chen L (1992). Seventh cervical nerve root transfer from the contralateral healthy side for treatment of brachial plexus root avulsion. J Hand Surg Br.

[41] Medical Research Council (1976). Aids to Examination of the Peripheral Nervous System. Memorandum No. 45.

[42] Yang LJ, Chang KW, Chung KC (2012). A systematic review of nerve transfer and nerve repair for the treatment of adult upper brachial plexus injury. Neurosurgery.

[43] Kim DH, Murovic JA, Tiel RL, Kline DG (2004). Mechanisms of injury in operative brachial plexus lesions. Neurosurg Focus.

[44] Estrella EP (2011). Functional outcome of nerve transfers for upper-type brachial plexus injuries. J Plast Reconstr Aesthet Surg.

[45] Narakas AO, Hentz VR (1988). Neurotization in brachial plexus injuries. Indication and results. Clin Orthop Relat Res.

[46] Sulaiman OAR, Boyd JG, Gordon T, Kettenmann H, Ransom BR (2004). Axonal regeneration in the peripheral nervous system of mammals. Neuroglia.

[47] Brown PW (1972). Factors influencing the success of the surgical repair of peripheral nerves. Surg Clin North Am.

[48] Furey MJ, Midha R, Xu QG, Belkas J, Gordon T (2007). Prolonged target deprivation reduces the capacity of injured motoneurons to regenerate. Neurosurgery.

[49] Kim DH, Cho YJ, Tiel RL, Kline DG (2003). Outcomes of surgery in 1019 brachial plexus lesions treated at Louisiana State University Health Sciences Center. J Neurosurg.

[50] Sulaiman OA, Gordon T (2000). Effects of short-and long-term Schwann cell denervation on peripheral nerve regeneration, myelination, and size. Glia.

[51] Hems T (2011). Nerve transfers for traumatic brachial plexus injury: advantages and problems. J Hand Microsurg.

[52] Kline DG, Hudson AR (2010). Nerve transfers for brachial plexus injuries. J Neurosurg.

[53] Carter PM, Poremski TM (2015). Nerve transfers in brachial plexus injury: a review of outcomes. J Plast Reconstr Aesthet Surg.

[54] Wirth MA, Foad MJ (2004). Brachial plexus reconstruction: outcomes of combining nerve transfers and grafting. J Shoulder Elbow Surg.

[55] Sardesai S, Lister G (2013). A comparative study of nerve transfers and nerve grafting in brachial plexus injuries. Plast Reconstr Surg.

[56] Siqueira MG, Martins RS, Solla D, Faglioni W, Foroni L, Heise CO (2019). Functional outcome of spinal accessory nerve transfer to the suprascapular nerve to restore shoulder function: Results in upper and complete traumatic brachial plexus palsy in adults. Neurol India.

[57] Texakalidis P, Tora MS, Lamanna JJ, Wetzel J, Boulis NM (2019). Combined Radial to Axillary and Spinal Accessory Nerve (SAN) to Suprascapular Nerve (SSN) Transfers May Confer Superior Shoulder Abduction Compared with Single SA to SSN Transfer. World Neurosurg.

[58] Bhandari PS, Sadhotra LP, Bhargava P, Bath AS, Mukherjee MK, Bhatti T, Maurya S (2009). Surgical outcomes following nerve transfers in upper brachial plexus injuries. Indian J Plast Surg.

[59] Garg R, Merrell GA, Hillstrom HJ, Wolfe SW (2011). Comparison of nerve transfers and nerve grafting for traumatic upper plexus palsy: a systematic review and analysis. J Bone Joint Surg Am.

[60] Chu B, Wang H, Chen L, Gu Y, Hu S (2016). Dual Nerve Transfers for Restoration of Shoulder Function After Brachial Plexus Avulsion Injury. Ann Plast Surg.

[61] Leechavengvongs S, Witoonchart K, Uerpairojkit C, Thuvasethakul P, Malungpaishrope K (2006). Combined nerve transfers for C5 and C6 brachial plexus avulsion injury. J Hand Surg Am.

[62] Bertelli JA, Ghizoni MF (2010). Nerve root grafting and distal nerve transfers for C5-C6 brachial plexus injuries. J Hand Surg Am.

[63] Uerpairojkit C, Leechavengvongs S, Witoonchart K, Malungpaishorpe K, Raksakulkiat R (2009). Nerve transfer to serratus anterior muscle using the thoracodorsal nerve for winged scapula in C5 and C6 brachial plexus root avulsions. J Hand Surg Am.

[64] Ayhan E, Soldado F, Fontecha CG, Bertelli JA, Leblebicioglu G (2020). Elbow flexion reconstruction with nerve transfer or grafting in patients with brachial plexus injuries: A systematic review and comparison study. Microsurgery.

[65] Teboul F, Kakkar R, Ameur N, Beaulieu JY, Oberlin C (2004). Transfer of fascicles from the ulnar nerve to the nerve to the biceps in the treatment of upper brachial plexus palsy. J Bone Joint Surg Am.

[66] Math RK, Lyons AB, Bietz G (2006). Physiological and clinical advantages of median nerve fascicle transfer to the musculocutaneous nerve following brachial plexus root avulsion injury. J Neurosurg.

[67] Kakinoki R, Ikeguchi R, Dunkan SF, Nakayama K, Matsumoto T, Ohta S (2010). Comparison between partial ulnar and intercostal nerve transfers for reconstructing elbow flexion in patients with upper brachial plexus injuries. J Brachial Plex Peripher Nerve Inj.

[68] Texakalidis P, Hardcastle N, Tora MS, Boulis NM (2020). Functional restoration of elbow flexion in nonobstetric brachial plexus injuries: A meta-analysis of nerve transfers versus grafts. Microsurgery.

[69] Xiao C, Lao J, Wang T, Zhao X, Liu J, Gu Y (2014). Intercostal nerve transfer to neurotize the musculocutaneous nerve after traumatic brachial plexus avulsion: a comparison of two, three, and four nerve transfers. J Reconstr Microsurg.

[70] Kline DG, Hudson AR, Kline DG, Hudson AR (1995). Stretch injuries to the brachial plexus. Nerve injuries, operative results for major nerve injuries, entrapments, and tumors.

[71] Chuang DC-C, Yeh M-C, Wei F-C (1992). Intercostal nerve transfer of the musculocutaneous nerve in avulsed brachial plexus injuries: evaluation of 66 patients. J Hand Surg.

[72] Ogino O, Naito T (1995). Intercostal nerve crossing to restore elbow flexion and sensibility of the hand for a root avulsion type of brachial plexus injury. Microsurgery.

[73] Merrell GA, Barrie KA, Katz DL, Wolfe SW (2001). Results of nerve transfer techniques for restoration of shoulder and elbow function in the context of a meta-analysis of the English literature. J Hand Surg Am.

[74] Barthel PY, Barbary S, Breton A, Apredoaei C, Dap F, Mansat P, Dautel G (2014). Restauration de la flexion du coude dans les paralysies traumatiques C5-C6 et C5-C6-C7 : étude bicentrique rétrospective comparant simple versus double neurotisation [Recovery of elbow flexion in post-traumatic C5-C6 and C5-C6-C7 palsy: retrospective dual-center study comparing single and double nerve transfer]. Chir Main.

[75] Carlsen BT, Kircher MF, Spinner RJ, Bishop AT, Shin AY (2011). Comparison of single versus double nerve transfers for elbow flexion after brachial plexus injury. Plast Reconstr Surg.

[76] Ray WZ, Pet MA, Yee A, Mackinnon SE (2011). Double fascicular nerve transfer to the biceps and brachialis muscles after brachial plexus injury: clinical outcomes in a series of 29 cases. J Neurosurg.

[77] Siqueira MG, Martins RS, Faglioni WF, Foroni L, Heise CO (2018). Restoration of elbow flexion in traumatic upper brachial plexus palsy in adults: outcome with intraplexus distal nerve transfers in 78 patients. Arq Bras Neurocirurg.

[78] Romeih M, Mazrou IA (2025). Comparing the long-term results of Oberlin II versus intercostal neurotization for elbowflexion restoration (Prospective study). Hand Surg Rehabil.

[79] Coulet B, Boretto JG, Lazerges C, Chammas M (2010). A comparison of intercostal and partial ulnar nerve transfers in restoring elbow flexion following upper brachial plexus injury (C5-C6+/-C7). J Hand Surg Am.

[80] Borschel GH, Clarke HM (2009). Obstetrical brachial plexus palsy. Plast Reconstr Surg.

[81] Malessy MJ, Pondaag W (2011). Nerve surgery for neonatal brachial plexus palsy. J Pediatr Rehabil Med.

[82] Lin JC, Schwentker-Colizza A, Curtis CG, Clarke HM (2009). Final results of grafting versus neurolysis in obstetrical brachial plexus palsy. Plast Reconstr Surg.

[83] Tse R, Kozin SH, Malessy MJ, Clarke HM (2015). International Federation of Societies for Surgery of the Hand Committee report: the role of nerve transfers in the treatment of neonatal brachial plexus palsy. J Hand Surg Am.

[84] Xu WD, Xu JG, Gu YD (2005). Comparative clinic study on vascularized and nonvascularized fulllength phrenic nerve transfer. Microsurgery.

